# Introgression of resistance to *Rhopalosiphum padi* L. from wild barley into cultivated barley facilitated by doubled haploid and molecular marker techniques

**DOI:** 10.1007/s00122-019-03287-3

**Published:** 2019-02-02

**Authors:** Inger Åhman, Therése Bengtsson

**Affiliations:** Department of Plant Breeding, Swedish University of Agricultural Sciences, P.O. Box 101, 230 53 Alnarp, Sweden

## Abstract

**Key message:**

Long-term pre-breeding using *Hordeum vulgare* ssp. *spontaneum* as a donor of bird cherry-oat aphid resistance has resulted in agronomically improved resistance sources of barley along with easy-to-use molecular markers.

**Abstract:**

Bird cherry-oat aphid (*Rhopalosiphum padi* L.) is a pest and a virus vector in barley to which there are no bred-resistant cultivars. The present study describes how resistance from *Hordeum vulgare* ssp. *spontaneum* has been introgressed in cultivated barley via five successive crosses with the same cultivar Lina (BC) and in parallel with other more modern barley cultivars. Most of the selections for resistance are based on measurements of individual aphid growth in the laboratory. This very slow phenotyping method has been complemented by molecular marker evaluation and application in part of the breeding material. Doubled haploid production in each generation has been crucial for more precise selection of lines with the quantitatively expressed resistance. A field trial of selected “BC_3_”-generation lines essentially confirmed the laboratory results, so did genotyping of the whole pedigree of parents and selected “BC_2_” and “BC_4_” offspring lines. The Infinium iSelect 50 K SNP assay confirmed relationships between lines and discerned several new markers for a resistance QTL on chromosome 2H.

**Electronic supplementary material:**

The online version of this article (10.1007/s00122-019-03287-3) contains supplementary material, which is available to authorized users.

## Introduction

Bird cherry-oat aphid (*Rhopalosiphum padi* L.) is a pest of small grain cereals in temperate regions worldwide (Blackman and Eastop 2007). Apart from the direct feeding damage that it causes, this aphid is also a vector of the harmful Barley Yellow Dwarf and Cereal Yellow Dwarf Viruses, BYDV/CYDV (Jarosova et al. 2016). Yield losses due to the combined infestation of aphids and BYDV/CYDV in winter barley can be as high as 80%, but field-to-field and year-to-year variation is large (Dedryver et al. 2010). Aphid and virus damage may be reduced by pesticide application, but access to efficient treatments against aphids begins to be limited due to product withdrawals and aphids becoming resistant to the control agents (Dewar and Foster 2017). Host resistance to aphids is an attractive alternative or complement to other control measures, and there are barley cultivars bred for resistance to Russian wheat aphid (RWA; *Diuraphis noxia)* and greenbug (GB; *Schizaphis graminum)*, even with combined resistance to both (Mornhinweg et al. 2012, 2017). As far as known there are no examples of breeding for resistance to *R. padi* leading to commercial varieties. The present study aims at developing agronomically improved resistance sources together with selection tools to be used in commercial barley breeding.

In cold temperate regions *R. padi* overwinters as eggs on *Prunus padus* L. from which females emerge in spring. After a couple of parthenogenetic wingless generations, winged females develop and migrate to grasses. In spring-sown cereals these migrants start colonies consisting of successive clonal aphid generations with a population peak after approximately 1 month. Plant resistance traits that reduce aphid fecundity during this period from seedling to beginning of ear emergence can have a profound effect on aphid population growth. A simulation study estimated that a 20% increase in aphid development time reduces the peak population size by more than 50% and a 20% reduction in aphid birth rate results in a 40% reduction in peak population size (Wiktelius and Pettersson 1985). Birth rate is related to adult size since small females carry few embryos (Dewar 1977). This is the rationale for using reduced nymphal growth for phenotyping host resistance to *R. padi* in the present study, since *R. padi* does not cause any conspicuous leaf symptoms possible to use as phenotypic markers for selecting resistant plant genotypes in breeding programs. Breeding for resistance to RWA and GB has been facilitated by typical leaf symptoms such as chlorosis (RWA and GB), leaf rolling (RWA) and plant death at high densities (GB), enabling selections based on plant symptoms, rather than aphid growth as in the present study. *R. padi* causes more subtle plant symptoms such as reduced plant growth and plant yellowing when aphid populations are dense.

Barley gene sources for resistance to *R. padi* have been reported (Porter et al. 1999), among which is the progenitor of barley, *Hordeum vulgare* ssp. *spontaneum* (Weibull 1994; Åhman et al. 2000; Ninkovic and Åhman 2009). In a previous unsuccessful attempt to use high gramine concentration as a resistance factor (Åhman et al. 2000) one out of several sources for high gramine concentration, an accession of *H. v.* ssp. *spontaneum*, was found to reduce aphid growth by more than 40% of that on common barley cultivars (Delp et al. 2009). This gene source was crossed with the susceptible cultivar Lina, and doubled haploid (DH) lines were developed and tested for resistance measured as reduced aphid growth compared to Lina. A quantitative trait loci (QTL) study of this population localized a QTL marker on the short arm of chromosome 2H explaining 23% of the variance in aphid weight (Louise O’Donoghue, personal communication).

The present study describes how this QTL marker was validated in DH barley populations and used as a complement to measuring aphid weight in a backcross (BC) breeding program to cultivar Lina. Selections in populations from successive crosses to other, more modern barley cultivars were made in parallel. Some of the selected BC_3_F_1_ DH lines were tested in the field with natural infestation of *R. padi*. Based on these results, one more round of BC, DH production and aphid resistance tests were performed. A 50-K SNP analysis of the whole pedigree of parents and selected offspring confirmed relationships and discerned several new markers for the resistance on chromosome 2H.

## Materials and methods

### Plant material

**Fig. 1:**
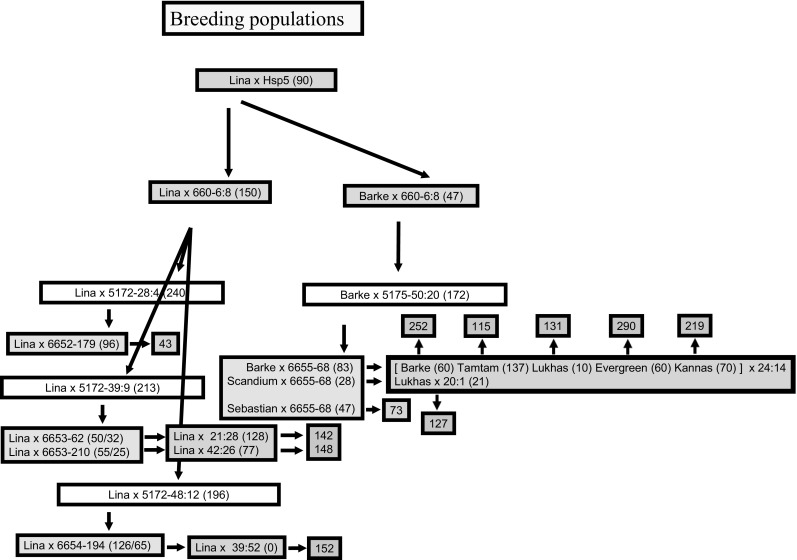
Pedigree for the breeding material. Parents of: F_1_ orange box, “BC_1_” gray boxes, “BC_2_” white boxes, “BC_3_” yellow boxes and “BC_4_” green boxes. The first number (before “-” or “:”) of a breeding line used as parent indicates from which population (sibling group) it was selected as being resistant. The number of the last population in a “BC” series is also indicated, in green (from “BC_3_”) or blue (from “BC_4_”) boxes. Numbers within parenthesis indicate how many lines from the cross that were tested with aphids: (*y*) = (number of DH lines tested with aphids) or (*x*/*y*) = (number of DH lines tested for QTL1 marker/number of DH lines with the QTL1 marker and those were all tested with aphids) (color figure online)

The aphid resistance source was a *Hordeum vulgare ssp. spontaneum* accession from Canada Park in Israel, here called Hsp5. Anther culture technique was used for DH production in F_1_, BC_1_F_1_ and BC_2_F_1_ (Åhman et al. 2000), whereas the DH lines from BC_3_F_1_ and BC_4_F_1_ were produced by microspore culture technique (http://www.nordicseed.dk/laboratoriet accessed June 1, 2018). The reason for using different methods of DH production was that access to such services changed over time. DH plants from tissue culture were propagated in 1.5- or 2-L pots in greenhouses, and perforated plastic bags were put on before flowering to prevent cross-pollination. In one series of BC generations the cultivar Lina was used as female parent (Fig. 1) aiming at near isogenic lines. In another series, new promising cultivars or breeding lines at the time for a new crossing were used as female parents. Such crosses are also called backcrosses even though the recurrent parent is not the same over the generations. For such “BC_1_,” the same aphid-resistant F_1_ line used as male parent in the cross with Lina was also used for a cross with the cultivar Barke. For BC_2_, three aphid-resistant BC_1_ lines were selected for another BC to Lina, and one of the lines from the cross with Barke was selected for another “BC” to Barke. For BC_3_, four BC_2_ lines were selected as male parents in BC to Lina and one for “BC” to cultivars Barke, Scandium and Sebastian. For “BC_4_” Barke, Tamtam, Lukhas, Evergreen and Kannas were used as female parents in crosses with two aphid-resistant “BC_3_F_1_” DH lines. BC_4_ to Lina involved three resistant BC_3_ lines (Fig. 1).

### QTL1 marker test

DNA was extracted from two 20-mm^2^-diameter fresh leaf disks of each seedling and genotype following the microextraction protocol by Cheung et al. (1993) when the F_1_, BC_1_ and BC_2_ DH lines were analyzed. DNA from BC_3_ lines was prepared from ca 1-cm pieces of seedling leaves dried by silica gel and thereafter grinded.

Previous mapping and QTL analysis for the aphid resistance trait “reduced aphid growth” had been performed at DNA Landmarks in Canada in the F_1_ population consisting of 90 DH lines. An ISSR marker, UBC856b, was associated with the QTL distally on chromosome 2HS. This marker is here called QTL1 marker and is located at 13.1 cM on the DNA Landmarks’ reference map (Cheung et al. 2010).

The primer used for the amplification of the UBC856b marker was ACACACACACACACACYA. The PCR was carried out in a final volume of 25 µl with 25 ng of genomic DNA, 0.2 µM of the ISSR primer, 1.5 mM of MgCl_2_, 1 × PCR buffer (20 mM Tris-Cl, pH 8.4, 50 mM KCl), 0.4 mM dNTPs (Invitrogen, Burlington, Canada), 2% formamide and 1.25 U of recombinant TAQ DNA Polymerase (Invitrogen, Burlington, Canada). The PCR conditions were as follows: 94 °C for 3 min, 45 cycles comprising denaturation at 94 °C for 15 s, annealing at 52 °C for 30 s and elongation at 72 °C at 90 s with a final extension at 72 °C for 5 min. Electrophoresis was performed on a 2% 3:1 NuSieve agarose gel (Mandel, Guelph, Canada) in 1 × TAE buffer.

### Aphid laboratory tests

Colonies of *R. padi* were reared on oats in glasshouse cages year-round, with minimum 16H light (natural light supplemented by 400-W HQIE lamps) and temperature minimum at 18 °C. New colonies were started every spring from migrants on the winter host *Prunus padus* L., ensuring that aphids were free of BYDV and that aphid genetic diversity was not eroded.

Estimations of barley seedling resistance to *R. padi* were based on nymphal growth in a standard assay (Åhman et al. 2000). Seeds were soaked with 0.75% H_2_O_2_ or pure water on filter paper in Petri dishes for 3 days in a refrigerator. After another 2–3 days in the laboratory, the germinating seeds were transplanted to potting soil in 10-cm-diameter plastic pots. For DH lines from F_1_, BC_1_F_1_ and BC_2_F_1_, slow-release-fertilized Hammenhög potting soil (1 dl of 15–4.8–10.8 NPK Osmocote Plus and 15 ml Micromax minerals added per 50 L) was used and for the following generations Emmaljunga potting soil without extra fertilizer. Each test included 20–25 test genotypes, including Lina as a control. A genotype was represented by four plants, each one placed in one of four white plastic trays (41 × 62 × 11 cm), with test genotypes in a random order. Cylindrical Perspex tubes (2 cm diameter, 5 cm high) were slipped over each seedling after another 2 days, allowing the plant to grow through. The trays were placed in a glasshouse chamber, with temperature set at 22 °C and photoperiod to minimum 16-h light by 400 W HQIE lamps supplementing daylight and 1 week later moved to a Conviron (E15, CMP 3244) growth cabinet under the same temperature and photoperiod conditions but with fluorescent lighting at 220 µmol photons/m^2^/s at plant level and minimum 80% RH. Part of the tests with the populations from BC_3_ and BC_4_ were performed in a walk-in growth chamber where it was space enough to grow test plants and make aphid tests simultaneously, under the same conditions as in the growth cabinet. The tests started 1 week after transplanting by adding 5 newborn nymphs to each tube cage which was then sealed with cotton wool at the top. The nymphs were offspring from alate females collected on the walls of the rearing cages the day before to reproduce on oats. After 4 days the nymphs were weighed singly on a Mettler M3 microbalance. The seedlings were then at BBCH stage 12–13 (Lancashire et al. 1991). To be able to compare between tests, mean aphid weight on the test genotype was divided by the mean weight of the aphids retrieved on the control Lina, separately for each of the four replicates. Lines with low mean aphid weights in this initial test were retested one or two times before final selection to be used as parents in new crosses. Also the resistance source Hsp5 was included in all tests, but here we show results in relation to the control Lina. Mean aphid weight on Hsp5 was 57.1% of that on Lina based on results from 25 tests (Delp et al. 2009).

### Aphid field test

The field experiment was performed at Lönnstorp (55°40′N, 13°06′E) in Lomma, southern Sweden, and included six selected “BC_3_F_1_” DH lines from the breeding populations and the four female parents. Experimental seed had been field propagated in Chile the preceding winter. Seeding rate, aiming at 350 plants per m^2^ in the field, was adjusted according to germination rate in a laboratory test. Eight of the genotypes were planted in six replicates, and two in four replicates due to lack of seed, in a randomized block design. Each 1-m^2^ plot was surrounded by 1-m bare ground. Plots consisted of 8 rows sown by hand in furrows prepared by a rake, on May 8, 2012. The experiment was surrounded by barley sown the day after, when also Yara 21 − 3 − 10 + 4S NPK mineral fertilizer was applied at a rate of 100 kg N per ha. The whole experimental plot was then covered by Agryl fleece to promote seed germination and to protect against bird damage. The fleece was removed on 22 May when the plants were in the 2–3 leaf stage (BBCH scale 12–13; Lancashire et al. 1991). A warm and calm period followed during which alate aphids infested the field. Weeds were managed by hand-hoeing.

Aphids were counted two times with 1-week interval, on 11–12 June and 18–20 June. On each occasion, number of *R. padi* was counted on 25 plants taken randomly from each plot. Number of shoots per plant was recorded, and number of aphids per shoot was calculated. Plot means of *R. padi* per shoot were log +1-transformed and used in the statistical analyses. *R. padi* was the predominating aphid species, but occasional individuals of *Sitobion avenae* (Fabricius) and *Metopolophium dirhodum* (Walker) were also observed. Plant developmental stage was recorded according to the BBCH scale (Lancashire et al. 1991). Plots were harvested manually, and seeds were threshed, cleaned and weighed. Ca 5 g seed from each plot was analyzed for water content, and dry seed weight per plot was calculated.

### Genotyping and analysis

All the parents in the pedigree (Fig. 1), representatives of susceptible and additional resistant “BC_2_F_1_” DH lines and “BC_4_F_1_” DH lines selected as resistant were genotyped by the SNP&SEQ Technology Platform, Uppsala (www.genotyping.se) using the 50 K Illumina Infinium iSelect genotyping array for barley with 44,040 working assays (Bayer et al. 2017). Leaf pieces (4 cm long) were taken from seedlings, freeze-dried for 2 days and homogenized in a Retsch Mixer Mill MM400 (Retsch GmbH, Haan, Germany) for 1 min at 30 Hz. To each sample, 550 µl of pre-heated lysis buffer (100 mM Tris, 20 mM EDTA, 1.4 M NaCl and 2% CTAB, pH 8.0) was added followed by incubation for 1 h at 52 °C and centrifugation at 16,100*g* for 15 min. DNA was extracted from 200 µl supernatant in a QIAcube HT extraction robot using a QIAamp 96 DNA QIAcube HT Kit (Qiagen, Hilden, Germany) utilizing a standard DNA extraction protocol provided by the supplier except for a small modification; RNAse A, to a final concentration of 0.1 mg/mL, was added in the elution buffer (AE) diluted 1:10 in water.

SNP data were analyzed using the FlapJack software (Milne et al. 2010). Out of the 44,040 working assays included in the SNP array, 32,519 were anchored to a POPSEQ position (Mascher et al. 2013). Out of these, 669 had a call rate below 95% and 13,646 were monomorphic for the included genotypes. No genotypes with a call rate below 95% were identified. The 18,204 remaining SNP markers were used for all subsequent analysis in Flapjack. Information regarding annotations for loci surrounding certain SNP markers was obtained by the “find markers” option at www.floresta.eead.csic.es/barleymap (accessed June 20, 2018) using the POPSEQ_2017 map (Mascher et al. 2013) and the Morex genome map.

### Statistical analyses

Chi-square analysis of QTL1 marker presence/absence and ANOVA with barley genotype and replicate as main effects for aphid field and laboratory data, and seed weight per plot were performed with the software STATISTICA v. 9.1 from Statsoft, so were analyses of correlations between field and laboratory data.

## Results

### Aphid growth data of the populations in the pedigree

The pedigree consists of two branches: a true backcrossing program to cultivar Lina (BC_4_) and crosses to other more modern barley cultivars made in parallel (Fig. 1). Aphid growth data are presented as a proportion of aphid weight on cultivar Lina after 4 days of nymph development. Distributions of aphid data in successive generations are shown in supplementary Figs. (S1–S7). The distributions of aphid growth data are unimodal, and DH lines in the tails of the distributions are hereafter called resistant or susceptible to *R. padi*. Selection of resistant parents for the following generation was made after retesting resistant lines one or two times. Resistant lines from DH plants with few progenies were avoided as parents. These are the reasons why not all parents are from the category of lines with the very lowest aphid weights in each population (Fig. S1–S7).

### Molecular QTL1 marker analyses

Three of the four aphid-resistant DH lines from the BC_1_F_1_ generation used as male parents in crossings in BC_2_ had the QTL1 marker on chromosome 2HS. Representatives for the most resistant and most susceptible lines were tested for the QTL1 marker after phenotyping the resulting BC_2_F_1_ DH populations for aphid growth. As expected, since the male parent 5172-28:4 lacked the QTL1 marker, none of its offspring had the marker (Table 1). The presence/absence of the QTL1 marker in offspring from male parent 5172-48:12 perfectly matched the resistant/susceptible categorization, whereas in offspring from male parent 5172-39:9 there was one out of six categorized as resistant that lacked QTL1, but none of the six in the susceptible category had it. All of these populations were true backcrosses to Lina. The population that resulted from two subsequent crosses with Barke, with 5175-50:20 as male parent in “BC_2_,” had two mismatches for expected presence or absence of the QTL1 marker among the 10 lines categorized as either resistant or susceptible. No further marker selections were performed in lines from subsequent crosses with other female parents than Lina, due to this somewhat lower precision in predicting which lines are resistant as in the case when Barke was the female parent in “BC_2_” (Table 1).

**Table 1:** Presence of the QTL1 marker in the BC_2_F_1_ populations of DH lines characterized as among the most resistant (R) and the most susceptible (S) to *R padi* in the respective populations. The first three populations were from backcrosses to Lina and the fourth from a “backcross” to Barke

Father in backcrosses	QTL1 marker	No of tested lines of R- and S-type	No. of lines with QTL1 marker	No. of lines without QTL marker
5172-28:4	No	8R 8S	0 0	8 8
5172-39:9	Yes	6R 6S	5 0	1 6
5172-48:12	Yes	4R 4S	4 0	0 4
5175-50:20	Yes	5R 5S	4 1	1 4

Three populations from BC_3_ to Lina were analyzed for the QTL1 marker. In populations 21, 39 and 42, 64.0%, 51.6% and 45.5% of the lines had the marker (Fig. 1). The ratios of populations 39 and 42 were not significantly different from the expected 1:1 ratio, whereas the ratio for the presence of QTL1 in population 21 was significantly higher (*χ*^2^ = 3.92, *p* = 0.0477). From these three populations only those lines having the marker were tested for reduced aphid growth. Proportion of lines having equal to or lower mean aphid weights than Lina was in population 21:65.6%, in 39:58.5% and in 42:76.0%. Unexpectedly, since no marker selection had been applied in the “BC_3_F_1_” populations originating from the cross with Barke in “BC_1_” and “BC_2_,” the corresponding proportions were as high as 81.9%, 96.4% and 74.5% for populations 20, 24 and 73, respectively. In the population 43 from BC_3_ to Lina with a male parent that lacked QTL1, only 37.5% of the lines had mean aphid weights lower than or equal to Lina (Fig. S1–S7).

### Aphid field test

From the BC to Lina, three BC_3_F_1_ DH lines (21:28, 42:26 and 43:82; with mean aphid laboratory weights of 83.2%, 73.1% and 75.2% of that on Lina) were tested in the field along with the recurrent female parent Lina. One of each aphid-resistant line from crosses where the female parent was a more modern cultivar was also included along with their female parent cultivars Barke (DH line 24:14; laboratory weight 54.1% of that on Lina), Scandium (DH line 20:1; laboratory weight 76,5%) and Sebastian (DH line 73:47; laboratory weight 63.1%). Aphid mean weights of the female parents Barke, Sebastian and Scandium in relation to that on Lina were 92.0%, 104.9% and 98.5%, respectively (ANOVA of mean absolute aphid weights per replicate: *F* = 0.73, *p* > 0.05 *df* = 3,9). Aphid populations were measured two times, the first time 3 weeks after the first aphid arrivals, at which time plants were at late tillering stage (BBCH 2.) and 1 week later when the developmental stages varied from late tillering, to stem elongation (BBCH 3.) to booting (BBCH 41). All the cultivars (Scandium, Sebastian, Barke and Lina) were then at stem elongation stage (BBCH 37–39). BC to Lina varied somewhat, 21:28 (BBCH 37–41), 42:26 (32–37) and 43:82 (39–41). Among the other three lines, the line with Barke as female parent, 24:14, was the latest of all, still at tillering stage (BBCH 2.), the line with Sebastian as female parent, 73:47, was the earliest (39–41) and the line with Scandium as female parent, 20:1, showed a bit larger plant developmental range (37–41).

**Fig. 2:**
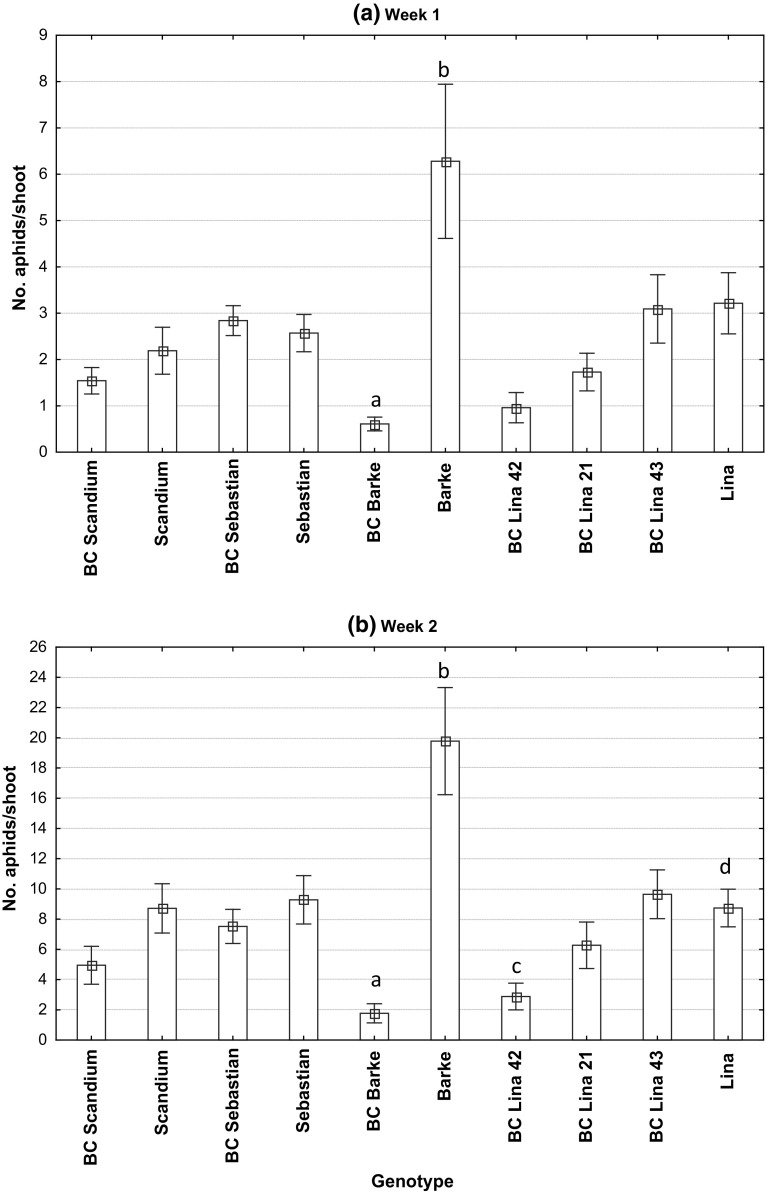
Mean number of *R. padi* per shoot based on samples of 25 plants per plot in six replicates (four in the case of BC Scandium and BC Lina 42) of “BC” breeding lines and their female parents during **a** week 1 (June 11–12, 2012) and **b** week 2 (18–20 June). Bar designations BC Lina 21, BC 42 and BC 43 correspond to lines 21:28, 42:26 and 43:82, BC Scandium to line 20:1, BC Sebastian to line 73:47 and BC Barke to line 24:14. Different letters above the bars of a breeding line and its female parent indicate significant differences in Tukey’s HSD test. Whiskers show standard error of least squared means

There were significant differences between barley genotypes in aphid numbers per tiller during both scorings (Fig. 2, week 1: *F* = 7.14, *p* < 0.001, *df* = 9.41; week 2: *F* = 17.28, *p* < 0.001, *df* = 9,41). Differences between replicates were also significant (week 1: *F* = 4.42, *p* < 0.01, *df* = 5,41; week 2: *F* = 11.17, *p* < 0.001, *df* = 5,41). In the first scoring, the line from the cross with Barke, 24:14, had significantly fewer *R. padi* per tiller than its female parent Barke (Tukey’s HSD test at *p* = 0.05; Fig. 2, week 1). In the second scoring also one of the Lina BC lines, 42:26, had significantly less aphids than its female parent Lina. There was the same tendency for the Lina BC line 21:28 and also the line 20:1 compared with its Scandium female parent, but the differences were not significant according to Tukey’s HSD test (Fig. 2, week 2). The Lina BC line 43:82 that lacked the QTL1 marker and the line with Sebastian as female parent, 73:47, did not have fewer aphids than their female parents (Fig. 2). Notable is that Barke had significantly higher aphid densities than all other genotypes in the second scoring. Number of aphids per plant and number of aphids per shoot were significantly, positively correlated (week 1: *r* = 0.97 and week 2: *r* = 0.95; *p* < 0.001, *df* = 8). Number of aphids per plant week 2 and log + 1 number of aphids per shoot week 2 were significantly, positively correlated with aphid weight relative to Lina in the laboratory test (*r* = 0.72 and *r* = 0.66, respectively, *p* < 0.05, *df* = 8).

There were significant differences between the barley genotypes in dry kernel weight per plot (*F* = 5.89, *p* < 0.001, *df* = 9,39; two samples discarded due to mixes at threshing), but in no case did the kernel yields differ significantly between the bred line and its female parent according to Tukey’s HSD test. Mean dry kernel weight per plot varied between 640 (42:26) and 809 g (Scandium).

### Genotyping the pedigree

**Fig. 3:**
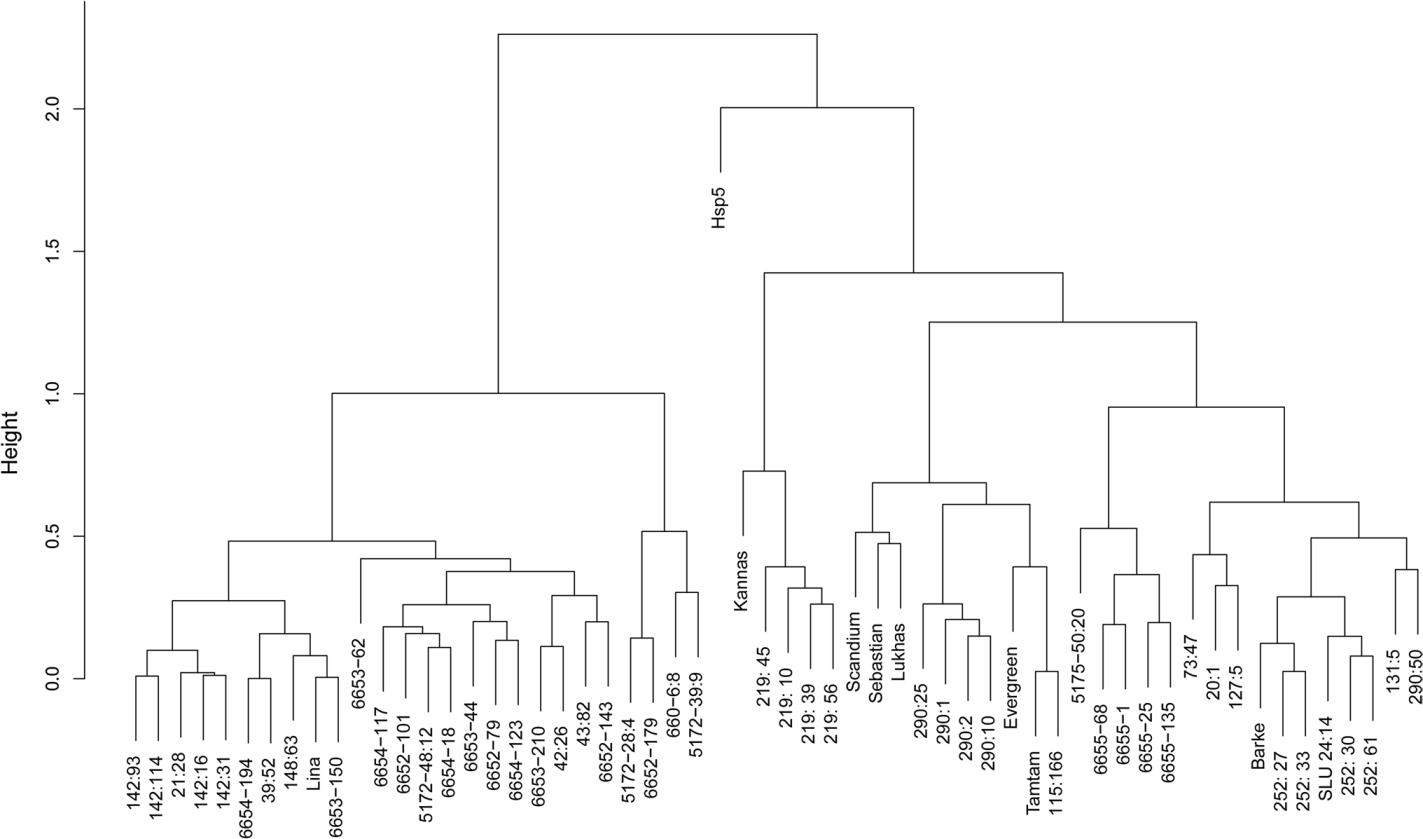
Genetic relationships between 58 barley genotypes based on SNP data from the 50 K Illumina Infinium iSelect genotyping array for barley comparing all chromosomes (18,204 mapped markers) using Flapjack software. The set consists of all the barley parents in the pedigree (Fig. 1), representatives of some susceptible and additional resistant “BC_2_F_1_” DH lines and “BC_4_F_1_” DH lines selected as resistant

**Fig. 4:**
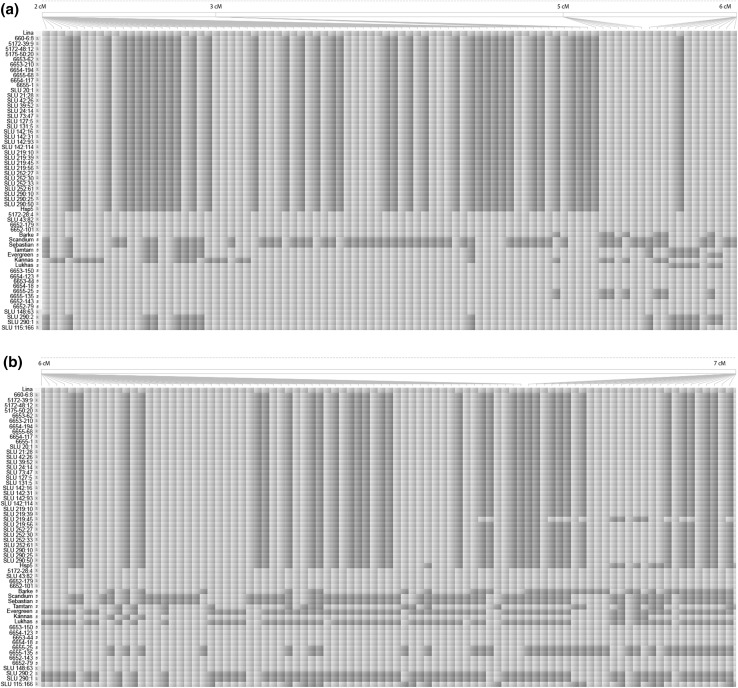
SNP at the distal end of chromosome 2HS analyzed with the 50 K Illumina Infinium iSelect genotyping array for barley. The color coding of SNP in cultivars and breeding lines is based on SNP similarity with cultivar Lina using Flapjack software. Green = the same nucleotide as Lina, red = different nucleotide compared to Lina. Lines or cultivars marked with white vertical bar and S = phenotyped as susceptible in aphid tests, with yellow bar and R = phenotyped as resistant (of which 5172-28:4, 6652-101, 6652-179 and 43:82 lack QTL1). **a** 0–6 cM and **b** 6–7 cM (color figure online)

All parents in the pedigree and selected offspring lines from BC_2_ and BC_4_ were SNP-genotyped and analyzed for genetic kinships with the 50 K Illumina Infinium iSelect SNP array. All the lines from BC to Lina clustered with Lina in one of the two main clusters (Fig. 3), confirming the pedigree information (Fig. 1). Parents and offspring lines often grouped together. Eighteen SNP distally (0–6 cM) on the short arm of chromosome 2H differed between the resistance source Hsp5 and all the susceptible parents (Fig. 4a). Annotations for loci surrounding these 18 SNP markers are listed in Tables S1 and S2. All parents selected as resistant, except the ones in the pedigree lacking QTL1, had the Hsp5 haplotype distally on 2HS. Eight “BC_2_F_1_” lines that had been phenotyped as susceptible had the same haplotype as their susceptible female parents Lina and Barke. Out of the 21 DH lines from “BC_4_” selected as resistant based on aphid growth, 17 had the Hsp5 SNP haplotype and four had the same haplotype as their susceptible female parents in the 0–6-cM interval (Fig. 4a). SNP indications of crossing over events started around 6–7 cM (Fig. 4b).

## Discussion

The difficulty to select for *R. padi* resistance together with the fact that confirmed resistance sources are very primitive from agronomic viewpoint (Weibull 1994; Porter et al. 1999; Åhman et al. 2000; Ninkovic and Åhman 2009) makes the road to produce an *R. padi*-resistant cultivar very long and costly. In the present study the resistance donor was wild barley, *H. v.* ssp. *spontaneum*, with many unfavorable traits, among them brittle rachis, long straw and need for vernalization. However, after a number of backcrosses to cultivated spring barley, many undesirable traits are removed and it is possible to compare plant performances in the field as was done in the “BC_3_F_1_” generation in 2012. That year, *R. padi* reached the economic threshold for pesticide application in 80% of the barley fields in the region (Holmblad et al. 2012). All the cultivars in our field study were well above the threshold, while the three breeding lines with the lowest aphid numbers per shoot were close to the threshold or below, depending on costs for pesticide application and barley price level (Aiéro et al. 2018). In particular one of the bred lines had much reduced aphid population size compared to the susceptible cultivars. But despite its much lower aphid pressure it did not outyield the cultivar that was recurrent parent in the last three successive crosses. Therefore, further breeding was needed and several of the resistant BC_4_F_1_ lines are now included in two commercial spring barley breeding programs along with the set of SNP markers on chromosome 2HS.

Overall, there was a significant correlation between laboratory aphid weight data and field aphid density data from the selected BC_3_F_1_ lines and their female parents, close to the time point when the *R. padi* populations peaked in the region (Holmblad et al. 2012). Compared to their susceptible female parents, two of the lines selected to reduce individual aphid growth had significantly less aphids per shoot and the tendency was the same for two more lines (but not for two others, one of which lacking the QTL1 marker). The parent/offspring combination Barke vs 24:14 with the largest difference in aphid densities exhibited a tenfold difference in number of aphids per shoot. The difference in aphid weight between this line and the control Lina in the laboratory test was just twofold, and aphids grow equally well on Lina and Barke in the laboratory (Ninkovic and Åhman 2009; and the present study). The discrepancy might merely be due to the longer exposure to host traits in the field, acting on successive aphid generations. The two lines with the smallest number of aphids, 24:14 and 42:26, were later in development than the other lines, something which might have affected aphid physiology negatively and thereby reduced aphid growth and reproduction more in the field. However, plant traits may influence not only aphid physiology but also aphid behavior. Mehrabi et al. (2016) found that aphid settling rate of apterous aphids confined for 2 days with selected lines from this breeding material was lower on resistant than susceptible genotypes in those cases where there were significant differences between such lines. This indicates that plant resistance traits influence host acceptance, which in turn may influence food intake, aphid growth and reproduction of successive aphid generations in the field. Unlike in the laboratory tests, there is also the additional host influence on aphid landing behavior in the field. Previous studies have shown that the denser the field stand the more immigrants are found, and the more green leaf area a plant has the more aphids land on it (Åhman et al. 1985). If this is the explanation for the greater difference in aphid population densities on Barke and 24:14 in the field than in aphid growth on the two barley genotypes in the laboratory, these barley genotypes must have differed a lot in this aspect when winged aphids arrived at seedling stage. However, for smaller plant biomass to be useful as a crop protection trait it must have an effect also in the absence of alternative hosts for aphids to land on, and it must not reduce yield. Likewise, it is negative from agronomic point of view if slow plant development is the cause for larger resistance effects in the field. Apart from such direct effects of host traits on the aphid as discussed above, there is also the possibility that natural enemies are responding to plant traits and thereby affect aphid densities differentially (Stenberg et al. 2015).

Volatile interactions between seemingly undamaged plants have been analyzed previously in part of this breeding material. Aphid host acceptance of Lina, 5172-28:4 and 5175-50:20 but not 5172-39:9 was reduced significantly after exposure to volatiles from the cultivar Alva known to be able to induce such antixenosis in several other cultivars, and Hsp5 was able to induce itself to lower aphid acceptance (Ninkovic and Åhman 2009). In the whole set of 19 barley genotypes included in that study, there was a correlation between the magnitude of plant volatile-induced antixenosis and aphid growth, tested independently. Whether the induced change in plant traits affects only host acceptance and feeding (antixenosis) and thereby reduce aphid growth or whether there are volatile-induced plant responses directly affecting aphid physiology and growth (antibiosis) is not known. Since there were 20 different genotypes or more, each represented by four plants per test occasion in our laboratory tests, some of the quantitative variation in aphid growth might be due to host volatile exposure varying from one test to another depending on barley genotype compositions. In addition, it is known that herbivore-attacked plants release volatiles that induce defense reactions in neighboring plants (Baldwin et al. 2006), but this effect has not been investigated in this particular plant material.

Molecular data confirmed the pedigree information, and SNP haplotype data distally on chromosome 2HS were in accordance with the phenotypic characterizations in 50 out of the 58 genotypes. Four out of the eight divergent lines were from BC_4_ and had their female parents’ haplotype distally on 2HS but were characterized as resistant. One of them was from a population (115) in which distribution of aphid growth data may indicate that the resistance was not transferred at crossing, possibly due to self-pollination. This particular breeding line was not given to barley breeders, a decision made before SNP information was available. The other four lines with low aphid weights in laboratory tests but with the susceptible haplotype in the SNP analysis of distal 2H were from the parent 5172-28:4 and subsequent populations in the pedigree lacking the QTL1 marker (i.e., 6652 and 43). A QTL study has been performed earlier in population 6652 from BC_2_, and a significant QTL was mapped on chromosome 3H, with resistance inherited from Lina and with no resistance QTL from Hsp5 (Cheung et al. 2010). One line from BC_3_F_1_ was selected for our field testing (43:82) and aphid density on that was not different from Lina’s. The ISSR marker for resistance QTL1 on chromosome 2H was less precise in the Barke than in the Lina genetic background when validated in BC_2_F_1_ DH lines, probably due to it being located less distally, at 13.1 cM (Cheung et al. 2010), than the SNP markers from the present study at 0–6 cM. Barley chromosomes 2H and 3H may harbor QTL for resistance to RWA and GB as well, but their locations differ from those conferring resistance to *R. padi* (Nieto-Lopez and Blake 1994; Mittal et al. 2008; Cheung et al. 2010; Tocho et al. 2012, 2013; Azhaguvel et al. 2014; Dahleen et al. 2015).

Part of the breeding material presented here has been analyzed for differences in constitutive and aphid-induced gene expressions. A microarray study identified candidate resistance genes by comparing two representatives of aphid-resistant genotypes (Hsp5 and 5172-28:4, without QTL1) with two aphid-susceptible cultivars (Lina and Kara; Delp et al. 2009). Applying strict criteria for gene regulation, just four genes were considered upregulated more in the resistant than in the susceptible ones. One of them, the protease inhibitor gene *CI2c*, was transformed to be overexpressed in barley, but this had no effect on *R. padi* (Losvik et al. 2018). When another of the four genes, *LOX 2.2*, was upregulated by transformation in barley, it influenced other genes in the jasmonate defense pathway and decreased short-term fecundity of the aphid (Losvik et al. 2017). In the 2HS locus at 0–6 cM there are several other candidate genes for *R. padi* resistance, among them R-genes coding for nucleotide-binding site leucine-rich repeat (NBS-LRR) proteins. Two cloned genes for aphid resistance are of that type, in tomato against biotypes of the potato aphid *Macrosiphum euphorbiae* (Kaloshian and Walling 2005) and in melon against *Aphis gossypii* (Boissot et al. 2016). Cysteine-rich receptor-like protein kinases also belong to the R-gene category (Sekhwal et al. 2015), and there is one such transcript known from the QTL region at 0–6 cM in our study. There is also an ethylene receptor gene. Transcripts associated with ethylene synthesis and signaling are well represented among differentially expressed hormonal-related transcripts in studies of *Arabidopsis thaliana* infested by phloem-feeding insects (Foyer et al. 2015). In wheat, ethylene signaling and certain MYB transcription factors contribute to resistance to the aphid *Sitobion avenae* via aphid-induced phloem occlusion (Zhai et al. 2017). On the contrary, MYB102 activates ethylene biosynthesis and promotes performance of *Myzus persicae*, the aphid species most commonly studied in interactions with *A. thaliana* (Zhu et al. 2018). Another transcript in the barley 0–6-cM 2HS region belongs to the NRT1 PTR family potentially involved in plant hormonal transport (Chiba et al. 2015). There is also a laccase gene in the 0–6-cM region in our study. Laccases are enzymes involved in polymerization of lignin, and overexpression of a laccase gene in cotton increased resistance to *A. gossypii,* whereas suppression increased cotton susceptibility to the aphid (Hu et al. 2018).

Technical development has been applied and contributed to the progress of this pre-breeding program for *R. padi* resistance during the 26-year-long period since the first crossing took place. The use of doubled haploid technique resulting in homozygous lines after each crossing has enabled replicated aphid experiments along with molecular marker analyses on the same barley genotypes. Molecular marker techniques have progressed from low marker densities to the now very dense SNP maps, at the same time with much reduced time needed for the technical analyses. In this particular example, one ISSR marker for *R. padi* resistance on 2HS is complemented with 18 optional easier-to-use SNP markers. However, the type of resistance gene and its exact position in this locus are still unknown. There is an ongoing attempt to capture R-genes and R-gene-like sequences in this breeding material which will hopefully reveal whether the resistance mechanism is of this kind or another.

### Author contribution statement

IÅ led the pre-breeding program since the start, performed part of the experiments, analyzed the aphid phenotyping data from laboratory and field and wrote the main part of the manuscript. TB performed the SNP analyses and wrote that part of the manuscript. Together IÅ and TB finalized the manuscript.

## Electronic supplementary material

Below is the link to the electronic supplementary material.

**Fig. S1** Distribution of aphid growth data in the successive barley populations from backcrosses to cultivar Lina ending with population 142 from BC_4_. *X*-axis: aphid weight on each DH line as a mean percentage of that on cultivar Lina after 4 days of nymph development. *Y*-axis: number of DH lines observed to belong to a certain aphid weight class. Line number for the line selected as male parent for the following cross is indicated above the bar corresponding to its weight class (PPTX 188 kb)

**Fig. S2** Distribution of aphid growth data in the successive barley populations from backcrosses to cultivar Lina ending with population 148 from BC_4_. *X*-axis: aphid weight on each DH line as a mean percentage of that on cultivar Lina after 4 days of nymph development. *Y*-axis: number of DH lines observed to belong to a certain aphid weight class. Line number for the line selected as male parent for the following cross is indicated above the bar corresponding to its weight class (PPTX 193 kb)

**Fig. S3** Distribution of aphid growth data in the successive barley populations from backcrosses to cultivar Lina ending with population 39 from BC_3_. (Population 152 has not yet been tested with aphids). *X*-axis: aphid weight on each DH line as a mean percentage of that on cultivar Lina after 4 days of nymph development. *Y*-axis: number of DH lines observed to belong to a certain aphid weight class. Line number for the line selected as male parent for the following cross is indicated above the bar corresponding to its weight class (PPTX 162 kb)

**Fig. S4** Distribution of aphid growth data in the successive barley populations from backcrosses to cultivar Lina ending with population 43 from BC_3_. *X*-axis: aphid weight on each DH line as a mean percentage of that on cultivar Lina after 4 days of nymph development. *Y*-axis: number of DH lines observed to belong to a certain aphid weight class. Line number for the line selected as male parent for the following cross is indicated above the bar corresponding to its weight class (PPTX 150 kb)

**Fig. S5** Distribution of aphid growth data in the successive barley populations from crosses with modern barley cultivars at the time for crossing, ending with populations 252, 115, 131, 290 and 219. *X*-axis: aphid weight on each DH line as a mean percentage of that on cultivar Lina after 4 days of nymph development. *Y*-axis: number of DH lines observed to belong to a certain aphid weight class. Line number for the line selected as male parent for the following cross is indicated above the bar corresponding to its weight class (PPTX 329 kb)

**Fig. S6** Distribution of aphid growth data in the successive barley populations from crosses with modern barley cultivars at the time for crossing, ending with population 127. *X*-axis: aphid weight on each DH line as a mean percentage of that on cultivar Lina after 4 days of nymph development. *Y*-axis: number of DH lines observed to belong to a certain aphid weight class. Line number for the line selected as male parent for the following cross is indicated above the bar corresponding to its weight class (PPTX 193 kb)

**Fig. S7** Distribution of aphid growth data in the successive barley populations from crosses with modern barley cultivars at the time for crossing, ending with population 73. *X*-axis: aphid weight on each DH line as a mean percentage of that on cultivar Lina after 4 days of nymph development. *Y*-axis: number of DH lines observed to belong to a certain aphid weight class. Line number for the line selected as male parent for the following cross is indicated above the bar corresponding to its weight class (PPTX 171 kb)

Supplementary material 8 (DOCX 39 kb)

Supplementary material 9 (DOCX 44 kb)

## Abbreviations

BBCH scale: Biologische Bundesanstalt, Bundessortenamt and CHemical industry scale
BC: Backcross
DH: Doubled haploid
GB: Greenbug
ISSR: Inter-simple sequence repeat
NBS-LRR: Nucleotide-binding site leucine-rich repeat
QTL: Quantitative trait locus
RWA: Russian wheat aphid
SNP: Single-nucleotide polymorphism
