# Supplementary figures and images for: Introgression of resistance to Rhopalosiphum padi L. from wild barley into cultivated barley facilitated by doubled haploid and molecular marker techniques
Source: Theor Appl Genet. 2019 Feb 2;132(5):1397–408. doi: 10.1007/s00122-019-03287-3 (PMC6477012; doi:10.1007/s00122-019-03287-3)

## Slide 1
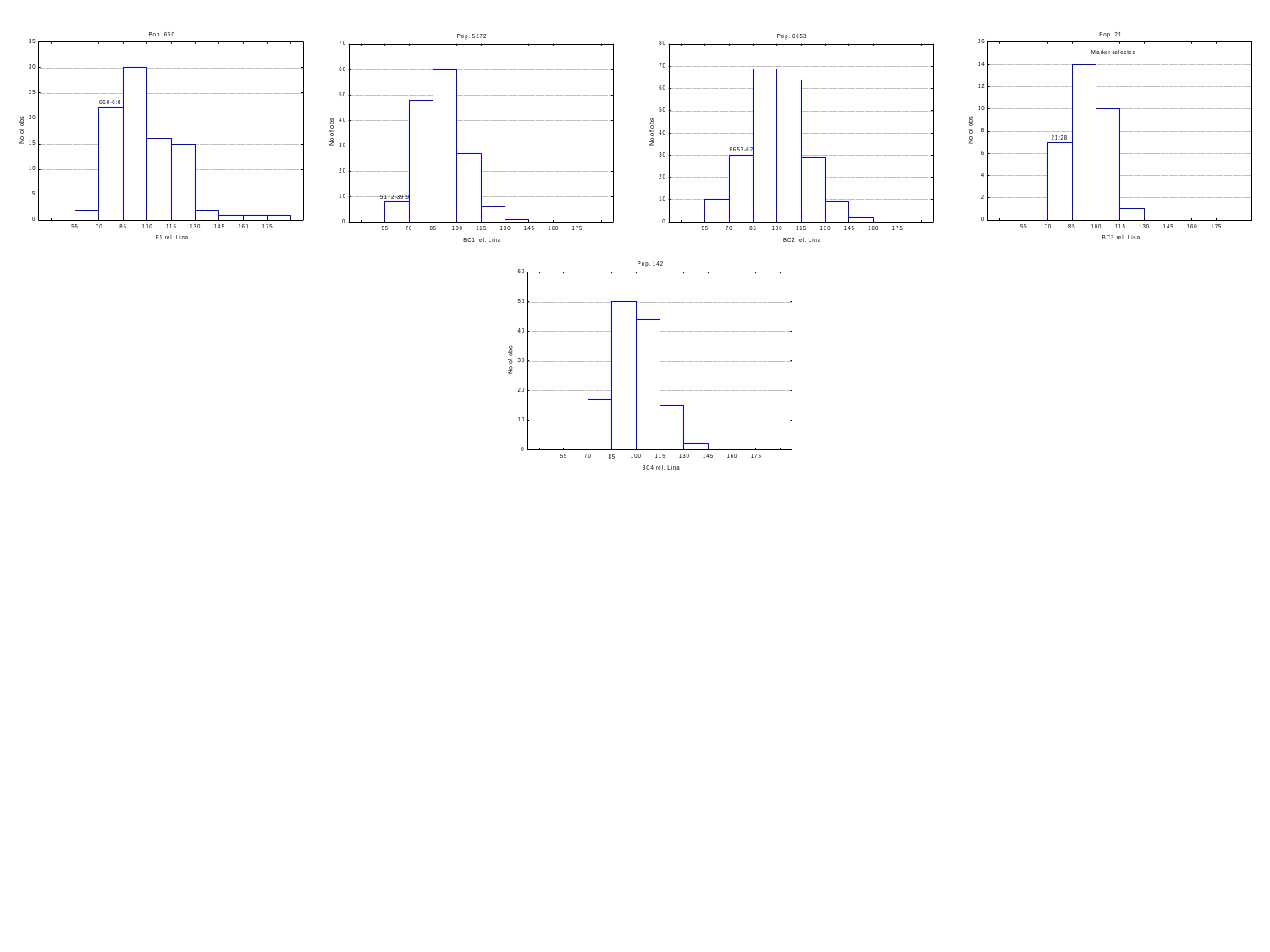

Supplement: Supplementary file 1 — Fig. S1 Distribution of aphid growth data in the successive barley populations from backcrosses to cultivar Lina ending with population 142 from BC4. X-axis: aphid weight on each DH line as a mean percentage of that on cultivar Lina after 4 days of nymph development. Y-axis: number of DH lines observed to belong to a certain aphid weight class. Line number for the line selected as male parent for the following cross is indicated above the bar corresponding to its weight class (PPTX 188 kb) [file 122_2019_3287_MOESM1_ESM.pptx]

## Slide 1
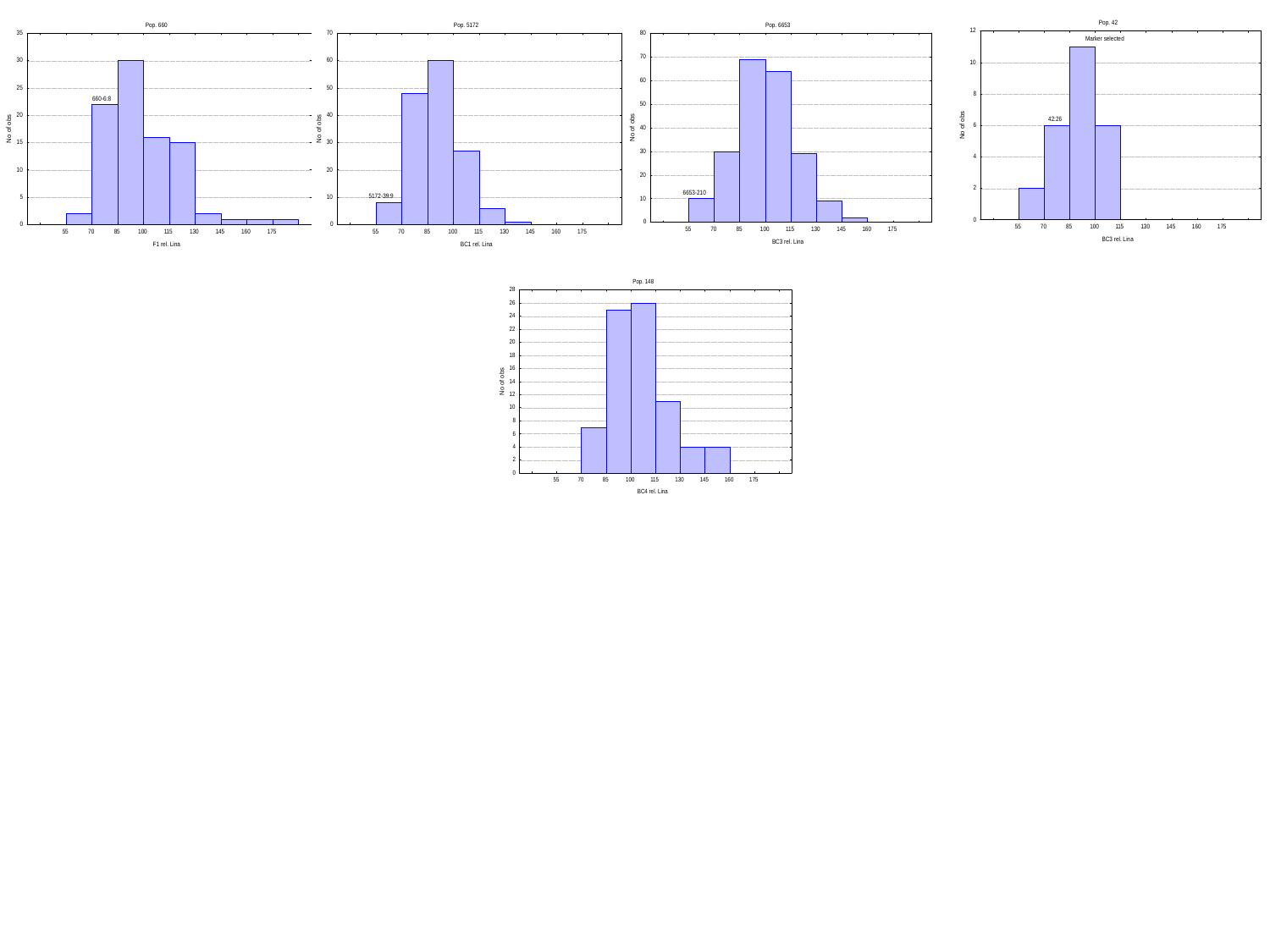

Supplement: Supplementary file 2 — Fig. S2 Distribution of aphid growth data in the successive barley populations from backcrosses to cultivar Lina ending with population 148 from BC4. X-axis: aphid weight on each DH line as a mean percentage of that on cultivar Lina after 4 days of nymph development. Y-axis: number of DH lines observed to belong to a certain aphid weight class. Line number for the line selected as male parent for the following cross is indicated above the bar corresponding to its weight class (PPTX 193 kb) [file 122_2019_3287_MOESM2_ESM.pptx]

## Slide 1
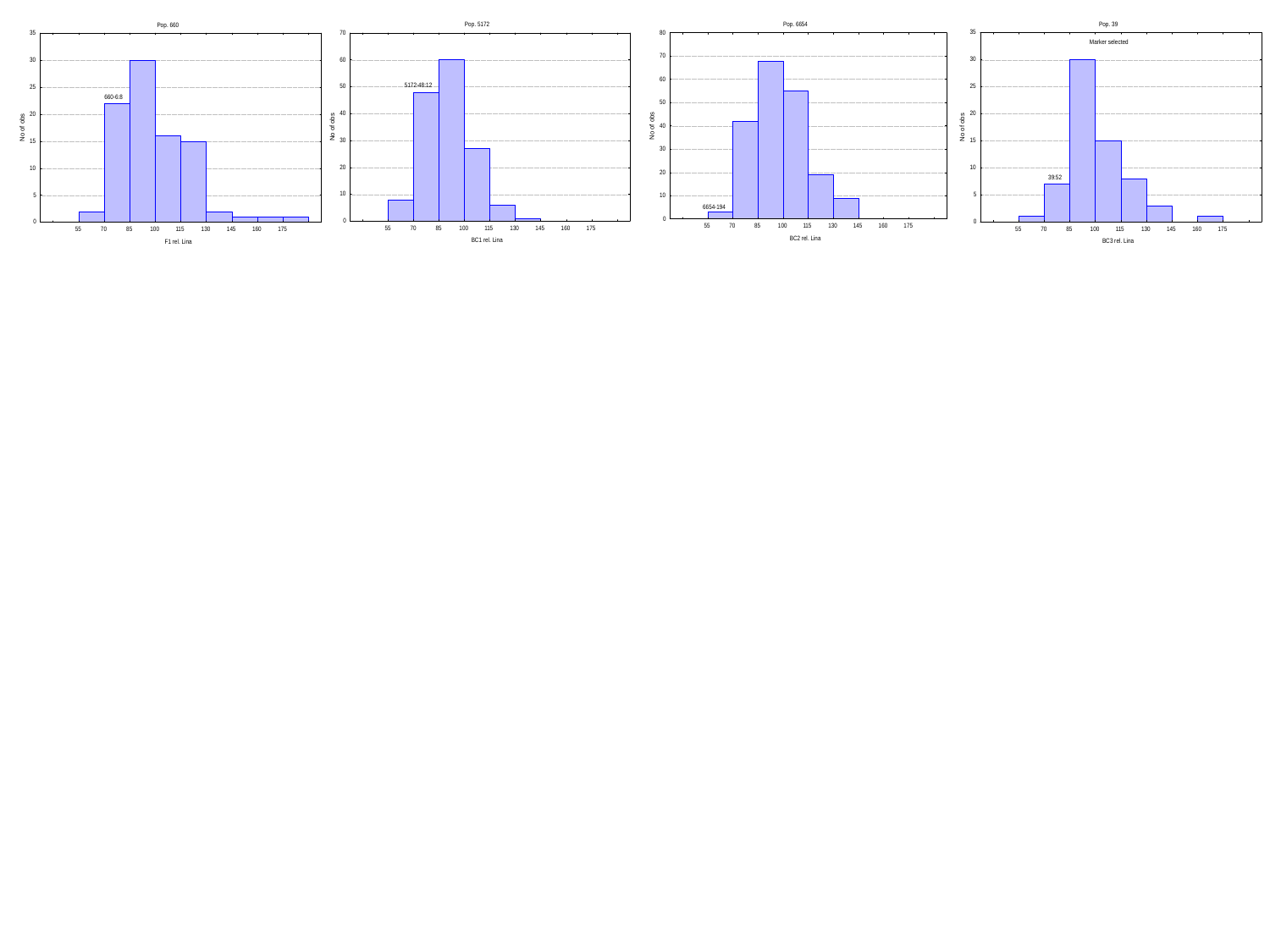

Supplement: Supplementary file 3 — Fig. S3 Distribution of aphid growth data in the successive barley populations from backcrosses to cultivar Lina ending with population 39 from BC3. (Population 152 has not yet been tested with aphids). X-axis: aphid weight on each DH line as a mean percentage of that on cultivar Lina after 4 days of nymph development. Y-axis: number of DH lines observed to belong to a certain aphid weight class. Line number for the line selected as male parent for the following cross is indicated above the bar corresponding to its weight class (PPTX 162 kb) [file 122_2019_3287_MOESM3_ESM.pptx]

## Slide 1
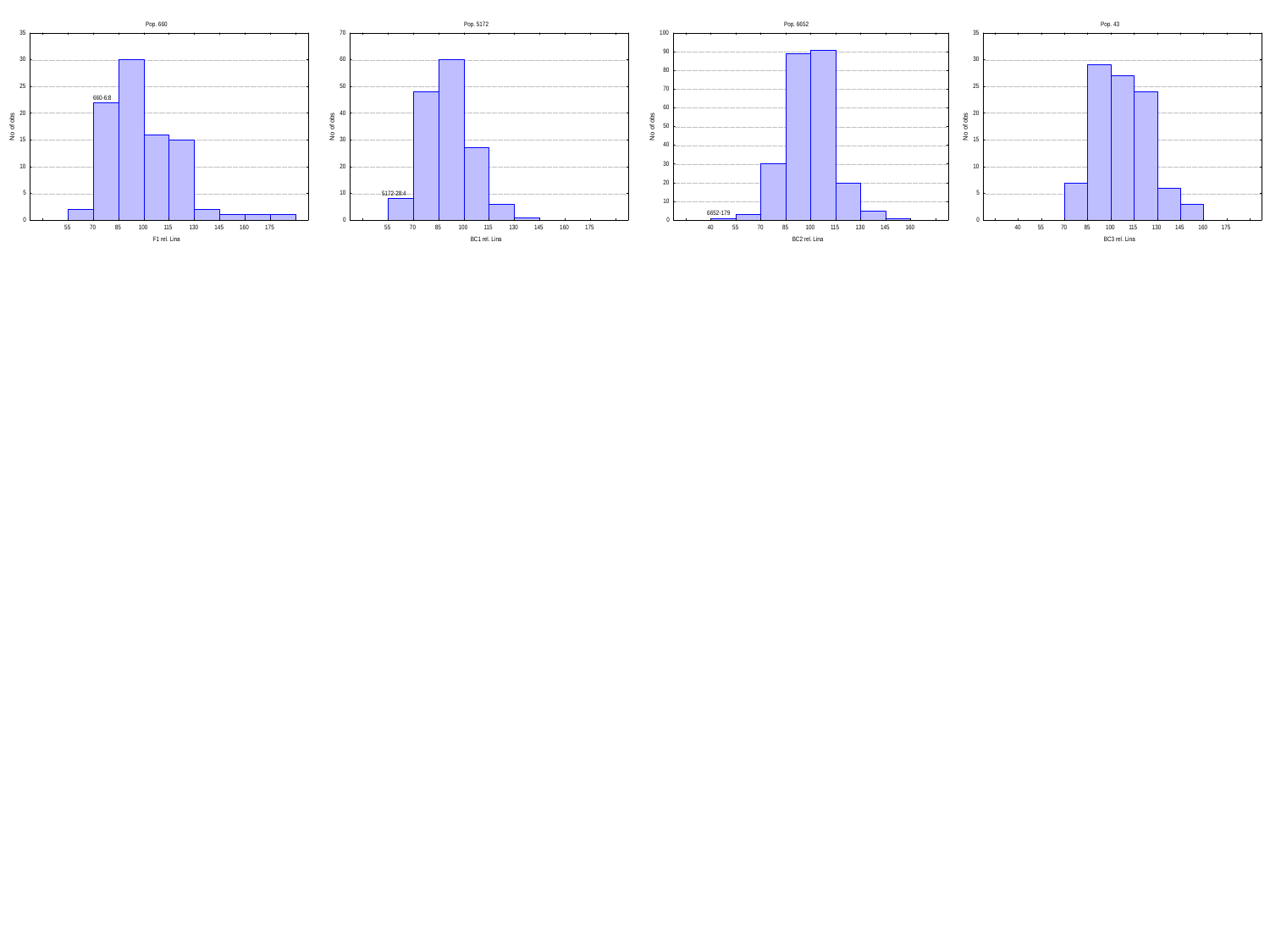

Supplement: Supplementary file 4 — Fig. S4 Distribution of aphid growth data in the successive barley populations from backcrosses to cultivar Lina ending with population 43 from BC3. X-axis: aphid weight on each DH line as a mean percentage of that on cultivar Lina after 4 days of nymph development. Y-axis: number of DH lines observed to belong to a certain aphid weight class. Line number for the line selected as male parent for the following cross is indicated above the bar corresponding to its weight class (PPTX 150 kb) [file 122_2019_3287_MOESM4_ESM.pptx]

## Slide 1
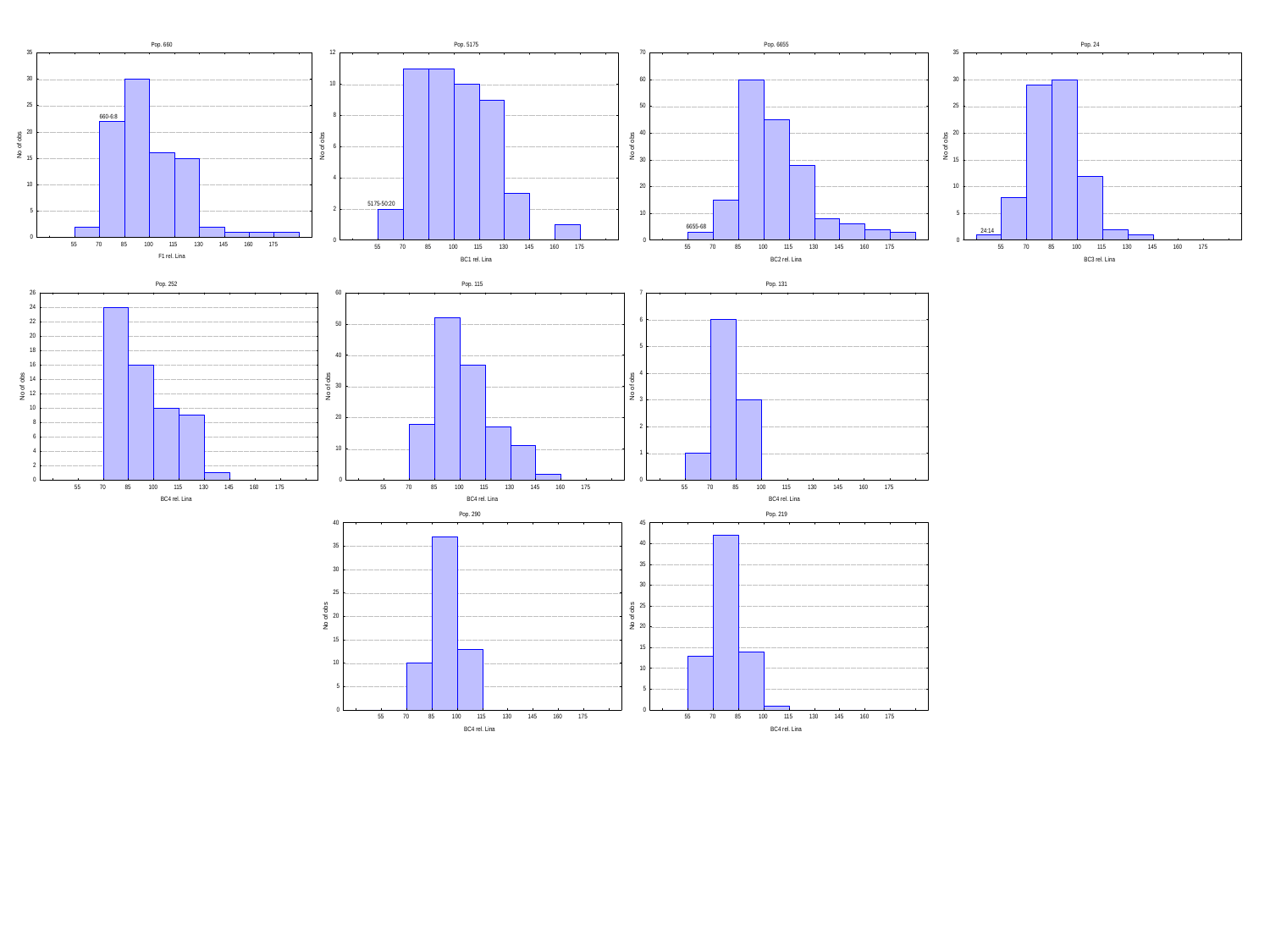

Supplement: Supplementary file 5 — Fig. S5 Distribution of aphid growth data in the successive barley populations from crosses with modern barley cultivars at the time for crossing, ending with populations 252, 115, 131, 290 and 219. X-axis: aphid weight on each DH line as a mean percentage of that on cultivar Lina after 4 days of nymph development. Y-axis: number of DH lines observed to belong to a certain aphid weight class. Line number for the line selected as male parent for the following cross is indicated above the bar corresponding to its weight class (PPTX 329 kb) [file 122_2019_3287_MOESM5_ESM.pptx]

## Slide 1
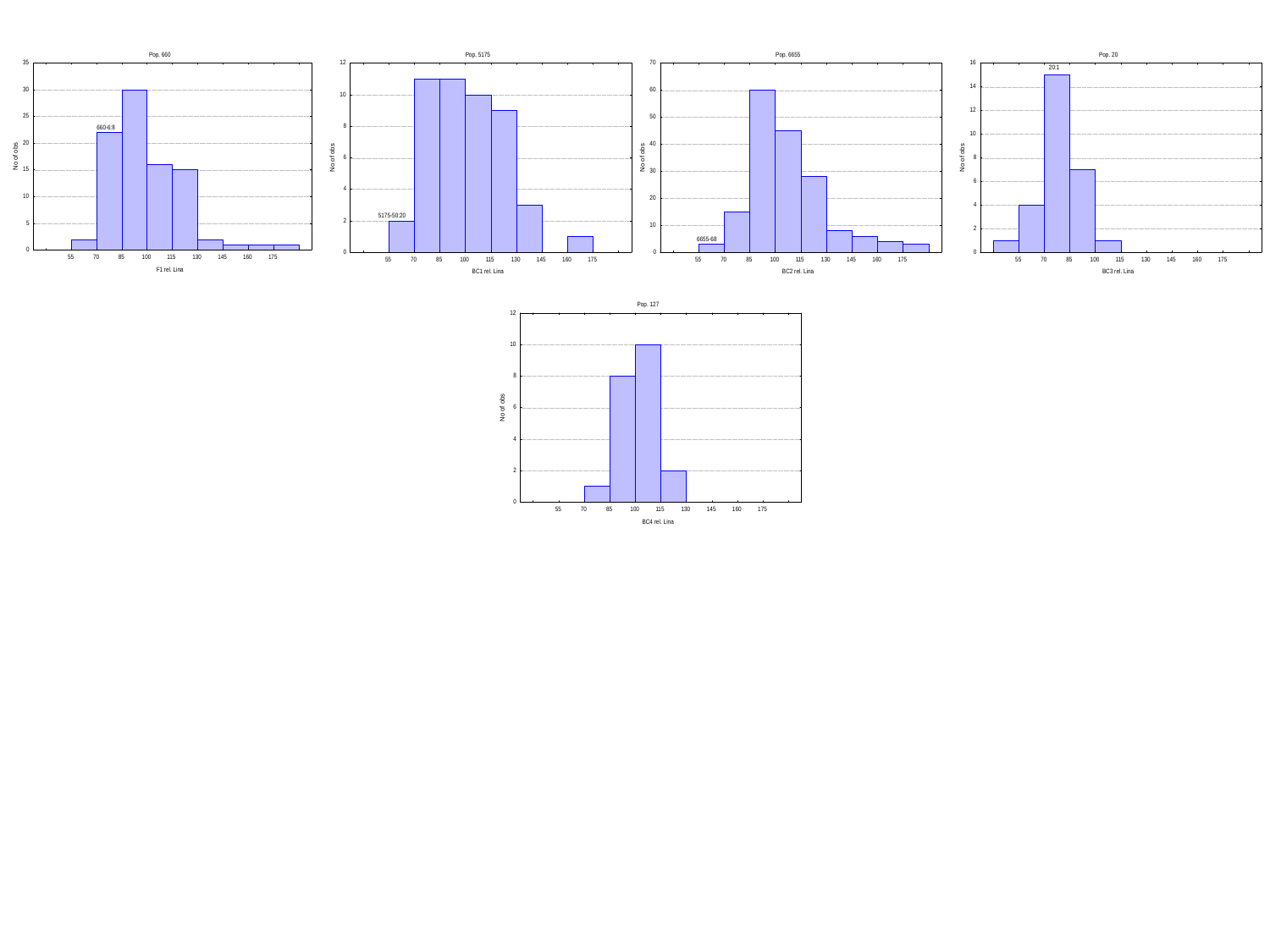

Supplement: Supplementary file 6 — Fig. S6 Distribution of aphid growth data in the successive barley populations from crosses with modern barley cultivars at the time for crossing, ending with population 127. X-axis: aphid weight on each DH line as a mean percentage of that on cultivar Lina after 4 days of nymph development. Y-axis: number of DH lines observed to belong to a certain aphid weight class. Line number for the line selected as male parent for the following cross is indicated above the bar corresponding to its weight class (PPTX 193 kb) [file 122_2019_3287_MOESM6_ESM.pptx]

## Slide 1
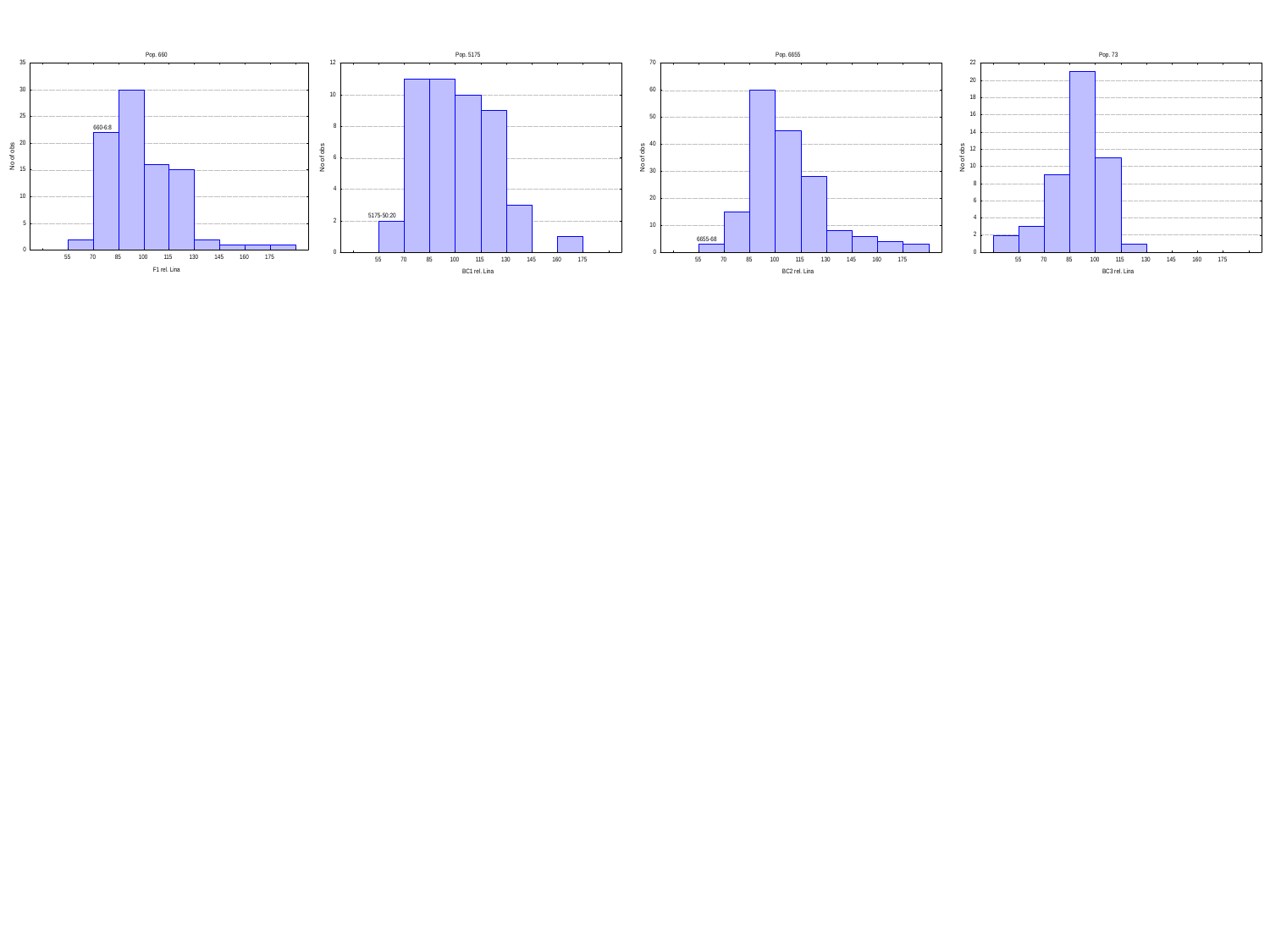

Supplement: Supplementary file 7 — Fig. S7 Distribution of aphid growth data in the successive barley populations from crosses with modern barley cultivars at the time for crossing, ending with population 73. X-axis: aphid weight on each DH line as a mean percentage of that on cultivar Lina after 4 days of nymph development. Y-axis: number of DH lines observed to belong to a certain aphid weight class. Line number for the line selected as male parent for the following cross is indicated above the bar corresponding to its weight class (PPTX 171 kb) [file 122_2019_3287_MOESM7_ESM.pptx]
