# Supplementary material for: Introgression of resistance to Rhopalosiphum padi L. from wild barley into cultivated barley facilitated by doubled haploid and molecular marker techniques
Source: Theor Appl Genet. 2019 Feb 2;132(5):1397–408. doi: 10.1007/s00122-019-03287-3 (PMC6477012; doi:10.1007/s00122-019-03287-3)
Supplement: Supplementary file 8 — Supplementary material 8 (DOCX 39 kb) [file 122_2019_3287_MOESM8_ESM.docx]

Supplementary Table S1 Details concerning the 18 SNP distally on 2HS that differed between the resistance source Hsp5 and all the susceptible parents. **Information from** [www.floresta.eead.csic.es/barleymap](http://www.floresta.eead.csic.es/barleymap) **(accessed 20 June 2018) based on the POPSEQ 2017 map (Mascher et al. 2013)**

| SNP | Chromosome | Position (cM) | Annotation of surrounding loci |
| --- | --- | --- | --- |
| **JHI-Hv50k-2016-58514** | 2H | 0.00 | ELM2 domain-containing protein, NBS-LRR disease resistance protein-like protein, Disease resistance protein (CC-NBS-LRR class) family, FAD-binding Berberine family protein, Cytochrome P450 family protein, Polycomb group protein EMBRYONIC FLOWER |
| **JHI-Hv50k-2016-58521** | 2H | 0.00 |  |
| **JHI-Hv50k-2016-58568** | 2H | 0.00 |  |
| **JHI-Hv50k-2016-58577** | 2H | 0.00 |  |
| **JHI-Hv50k-2016-59367** | 2H | 0.00 |  |
| **SCRI_RS_166806** | 2H | 0.00 |  |
| **SCRI_RS_219333** | 2H | 0.00 |  |
| **SCRI_RS_225720** | 2H | 0.00 |  |
| **HI-Hv50k-2016-58622** | 2H | 1.98 | ER to Golgi transport protein Yif1, Retrotransposon protein, putative, unclassified, NBS-LRR resistance-like protein |
| **JHI-Hv50k-2016-60256** | 2H | 2.12 | Cytochrome P450, Major Facilitator Superfamily protein, expressed, Unknown protein, NBS-LRR disease resistance protein homologue, C2H2-type zinc finger protein, Receptor protein kinase-like protein, Polyketide cyclase/dehydrase and lipid transport-like protein, ABC transporter B family member, Serpin-like protein, tRNA-dihydrouridine synthase, putative, Plant protein of unknown function (DUF827) |
| **JHI-Hv50k-2016-60352** | 2H | 2.12 |  |
| **HI-Hv50k-2016-59966** | 2H | 2.20 | B3 domain-containing protein, NBS-LRR disease resistance protein homologue, unknown protein, Late embryogenesis abundant (LEA) hydroxyproline-rich glycoprotein family , Cytochrome P450 family protein, expressed, Pentatricopeptide repeat protein, Lipid A export ATP-binding/permease protein MsbA |
| **JHI-Hv50k-2016-60002** | 2H | 2.20 |  |
| **JHI-Hv50k-2016-60088** | 2H | 2.20 |  |
| **JHI-Hv50k-2016-61865** | 2H | 4.59 | Laccase, putative |
| **JHI-Hv50k-2016-61868** | 2H | 4.59 |  |
| **JHI-Hv50k-2016-61871** | 2H | 4.59 |  |
| **JHI-Hv50k-2016-61823** | 2H | 5.38 | Cc-nbs-lrr resistance protein, Cytochrome P450 |
