# Supplementary material for: Introgression of resistance to Rhopalosiphum padi L. from wild barley into cultivated barley facilitated by doubled haploid and molecular marker techniques
Source: Theor Appl Genet. 2019 Feb 2;132(5):1397–408. doi: 10.1007/s00122-019-03287-3 (PMC6477012; doi:10.1007/s00122-019-03287-3)
Supplement: Supplementary file 9 — Supplementary material 9 (DOCX 44 kb) [file 122_2019_3287_MOESM9_ESM.docx]

Supplementary Table S2 Details concerning the 18 SNPs (in bold) distally on 2HS that differed between the resistance source Hsp5 and all the susceptible parents. **Information from** [www.floresta.eead.csic.es/barleymap](http://www.floresta.eead.csic.es/barleymap) **(accessed 20 June 2018) based on the Morex genome map (IBSC. 2012)**

| **Marker** | **Start** | **End** | **Strand** | **Other alignments** | **Gene class** | **Description** | **InterPro** | **GeneOntologies** | **PFAM** |
| --- | --- | --- | --- | --- | --- | --- | --- | --- | --- |
| HORVU2Hr1G000010 | 24516 | 32449 | + | No | HC_G | Polycomb group protein EMBRYONIC FLOWER 2 | IPR019135 | - | PF09733 |
| **JHI-Hv50k-2016-58514** | 26308 | 26309 | + | No |  |  |  |  |  |
| **SCRI_RS_166806** | 26308 | 26309 | + | No |  |  |  |  |  |
| **JHI-Hv50k-2016-58521** | 27431 | 27432 | + | No |  |  |  |  |  |
| HORVU2Hr1G000030 | 33881 | 37712 | - | No | HC_G | Ethylene receptor 2 | IPR003661,IPR005467,IPR011006,IPR014525,IPR029016,IPR001789,IPR003018,IPR003594 | GO:0005515,GO:0005789,GO:0007165,GO:0010105,GO:0038199,GO:0050896,GO:0051740,GO:0000155,GO:0000160,GO:0004673 | PF00072,PF01590 |
| **SCRI_RS_219333** | 35977 | 35978 | + | No |  |  |  |  |  |
| **SCRI_RS_225720** | 37521 | 37522 | + | No |  |  |  |  |  |
| HORVU2Hr1G000040 | 131031 | 135245 | + | No | HC_G | Histone deacetylase complex subunit SAP30 | IPR025718 | GO:0005515 | PF13867 |
| **JHI-Hv50k-2016-58568** | 134576 | 134577 | + | No |  |  |  |  |  |
| HORVU2Hr1G000050 | 139078 | 149044 | - | No | HC_G | NADPH-dependent codeinone reductase 1-5 | - | - | - |
| **JHI-Hv50k-2016-58577** | 139375 | 139376 | + | No |  |  |  |  |  |
| HORVU2Hr1G000090 | 202388 | 213168 | + | No | HC_G | Protein phosphatase 2C family protein | IPR001932 | GO:0003824 | PF00481 |
| HORVU2Hr1G000110 | 212267 | 212735 | - | No | LC_u | undescribed protein | - | - | - |
| **JHI-Hv50k-2016-58622** | 212492 | 212493 | + | No |  |  |  |  |  |
| HORVU2Hr1G000450 | 1211634 | 1218154 | - | No | HC_G | Cysteine-rich receptor-like protein kinase 41 | IPR002035 | - | - |
| **JHI-Hv50k-2016-59367** | 1215357 | 1215358 | + | No |  |  |  |  |  |
| HORVU2Hr1G001030 | 2198618 | 2240083 | + | No | HC_u | undescribed protein | - | - | - |
| **JHI-Hv50k-2016-59966** | 2199702 | 2199703 | + | No |  |  |  |  |  |
| HORVU2Hr1G001070 | 2245699 | 2246897 | + | No | HC_G | Late embryogenesis abundant (LEA) hydroxyproline-rich glycoprotein family | IPR004864 | - | PF03168 |
| **JHI-Hv50k-2016-60002** | 2246607 | 2246608 | + | No |  |  |  |  |  |
| HORVU2Hr1G001160 | 2374059 | 2377707 | + | No | HC_G | Cytochrome P450 superfamily protein | IPR001128,IPR002401,IPR017972 | GO:0055114,GO:0005506,GO:0016705,GO:0020037 | PF00067 |
| **JHI-Hv50k-2016-60088** | 2377500 | 2377501 | + | No |  |  |  |  |  |
| HORVU2Hr1G001320 | 2790022 | 2796496 | - | No | HC_G | Protein NRT1/ PTR FAMILY 8.5 | IPR000109,IPR020846 | GO:0005215,GO:0006810,GO:0016020 | PF00854 |
| CCJ47265.1 | 2794947 | 2795645 | - | No | ncbi\|HvPTR1-like_22 | putative proton-dependent olig | - | - | - |
| **JHI-Hv50k-2016-60256** | 2795156 | 2795157 | + | No |  |  |  |  |  |
| HORVU2Hr1G001360 | 2847952 | 2853677 | - | No | HC_G | tRNA-dihydrouridine(47) synthase [NAD(P)(+)]-like | IPR018517,IPR000571,IPR001269,IPR013785 | GO:0046872,GO:0050660,GO:0055114,GO:0003824,GO:0008033,GO:0017150 | PF01207 |
| **JHI-Hv50k-2016-60352** | 2848265 | 2848266 | + | No |  |  |  |  |  |
| HORVU2Hr1G002440 | 5116019 | 5428753 | + | No | HC_G | laccase 7 | IPR011707,IPR001117,IPR008972,IPR011706 | GO:0005507,GO:0016491,GO:0055114 | PF00394,PF07731,PF07732 |
| HORVU2Hr1G002500 | 5332106 | 5337834 | - | No | HC_G | HXXXD-type acyl-transferase family protein | IPR003480,IPR023213 | GO:0016747 | PF02458 |
| **JHI-Hv50k-2016-61823** | 5332555 | 5332556 | + | No |  |  |  |  |  |
| HORVU2Hr1G002600 | 5689544 | 5692391 | + | No | HC_G | Inosine-5'-monophosphate dehydrogenase | IPR000644 | - | PF00571 |
| **JHI-Hv50k-2016-61865** | 5691461 | 5691462 | + | No |  |  |  |  |  |
| **JHI-Hv50k-2016-61868** | 5691951 | 5691952 | + | No |  |  |  |  |  |
| **JHI-Hv50k-2016-61871** | 5692379 | 5692380 | + | No |  |  |  |  |  |
